# No “One-Size-Fits All”: chronic “Carryover” diagnoses dilute antibiotic prescribing rates for sinusitis among adults in primary and urgent care settings

**DOI:** 10.1017/ice.2024.200

**Published:** 2025-02

**Authors:** Mary Smith, Marten Hawkins, Chananid Laikijrung, Emily Mui, William Alegria, Thomas Leung, Alex Zimmet, David Ha, Marisa Holubar

**Affiliations:** 1 Department of Medicine, Division of Infectious Diseases and Geographic Medicine, Stanford University School of Medicine, Stanford, CA, USA; 2 Stanford Health Care, Stanford, CA, USA; 3 Department of Pharmacy, University of California, San Francisco, CA, USA; 4 Department of Pharmacy, Kaiser Permanente East Bay, Oakland, CA, USA

## Abstract

International Classification of Diseases, Tenth Revision (ICD-10) billing data used in outpatient stewardship metrics is under-described for acute and chronic sinusitis. We found that different sinusitis ICD-10 definitions impacted antibiotic prescribing rates (APRs). Chronic sinusitis ICD-10s dilute overall sinusitis APR, particularly in primary care settings and should be examined separately.

## Introduction

Sinusitis (acute and chronic) is a common infection in primary care, accounting for nearly 30 million diagnoses annually and >10% of antibiotic related visits.^1,2^ Despite national guidelines promoting judicious antibiotic use, management practices differ widely, highlighting sinusitis as an important antimicrobial stewardship target for optimizing outpatient prescribing.^3^

International Classification of Diseases, Tenth Revision (ICD-10) encounter coding has become foundational for outpatient stewardship metrics that identify suboptimal outpatient antibiotic use.^4,5^ While several studies have demonstrated the utility of the antibiotic prescribing rate (APR), a billing data-based antibiotic stewardship metric, few have evaluated it’s value for diagnoses such as sinusitis in which antibiotics are situationally indicated.^6,7^ We assessed the impact of different ICD-10 definitions on outpatient sinusitis APR to characterize it’s use and utility as a stewardship metric for these diagnoses.

## Methods

We included all adult telemedicine and office encounters from January 2021 to March 2022 categorized as sinusitis encounters by associated ICD-10s (Supplement Table 1) from two academic urgent care and eight primary care clinics. To identify these encounters, we used an internal data model to categorize most discharge encounter diagnoses by ICD-10 into tiers—those for which antibiotics are almost always (tier 1), sometimes (tier 2), or almost never (tier 3) indicated.^5–7^ All sinusitis ICD-10s are considered Tier 2. In the case of encounters with multiple ICD-10s, we classified the encounter by the ICD-10 with the lowest tier.^6^ For example, if an encounter was associated with both a Tier 1 (lowest tier) ICD-10 and a sinusitis ICD-10 (tier 2), it would be classified as a Tier 1 encounter and not included in this analysis to increase the likelihood that the associated antibiotic prescription was intended for sinusitis. We extracted encounter date, location, ICD-10, and antibiotic prescriptions from the electronic health record.

We calculated sinusitis APRs by dividing the number of encounters associated with an antibiotic by the total number of encounters. Sinusitis encounters were defined as acute or chronic based on billed ICD-10 code (Supplemental Table 1). We compared overall, acute, and chronic sinusitis APRs by clinic type to assess the impact of ICD-10 definition on APR.

We randomly selected 50% of chronic sinusitis encounters in 2021 as well as 100 acute sinusitis encounters for manual chart review to assess symptom documentation and if antibiotic prescriptions were adherent to institutional guidelines adapted from national guidelines for sinusitis.^8,9^ Antibiotic prescriptions were considered guideline-adherent if the patient met diagnostic criteria for persistent, worsening, or severe sinusitis, all conditions for which antibiotics were indicated in these guidelines. Antibiotics prescribed for chronic sinusitis encounters were deemed guideline adherent if documentation met criteria for acute sinusitis. Antibiotic choice and duration were not considered. This project was deemed nonhuman subjects research.

## Results

We included 987 total sinusitis encounters (507 urgent care, 480 primary care) from 821 unique patients with duplicate patients each having more than one unique encounter (Table 1.) Overall, acute sinusitis ICD-10s accounted for 673/987 (68.2%) and chronic sinusitis for 314/987 (31.8%). Acute sinusitis ICD-10s were used more commonly in urgent care settings (424/507, 83.6%) compared to primary care clinics (249/480, 51.9%).

**Table 1.** Antibiotic prescribing rate for sinusitis ICD-10s (1/2021–3/2022)

	Acute sinusitis^a^	Chronic sinusitis	All sinusitis
Primary care, n (APR %)	249 (65.5%)	231 (10.4%)	480 (39.0%)
Urgent care, n (APR %)	424 (76.9%)	83 (54.2%)	507 (73.2%)
Total combined, n (APR%)	673 (72.7%)	314 (22.0%)	987 (56.5%)

APR, antibiotic prescribing rate.

a Acute ICD-10s: J01.00, J01.01, J01.10, J01.11, J01.20, J01.21, J01.30, J01.31, J01.40, J01.41, J01.80, J01.81, J01.90, J01.91. Chronic ICD-10s: J32.0, J32.1, J32.2, J32.4, J32.8, J32.9.

The overall sinusitis APR was 56.5% (39.0% in primary care and 73.2% in urgent care clinics) (Table 1). The acute sinusitis APR was higher than chronic sinusitis APR (72.7% vs 22.0%), with more pronounced differences in primary care (65.5% vs 10.4%) compared to urgent care (76.9% vs 54.2%) (Table 1).

We performed manual chart review for 182 primary care and 100 urgent care patient encounters. We found that 42.3% of primary care sinusitis encounters had no documented active symptoms or did not address sinusitis during the encounter (n=77). Most of these encounters were associated with chronic sinusitis ICD-10s (Table 2). Alternatively, only 3% of urgent care sinusitis encounters did not document active symptoms (n=3) (Table 2).

**Table 2.** Urgent and primary care clinics adult sinusitis encounters—manual chart review (1/2021–12/2021)

	Sinusitis encounters, n	Total encounters without active symptoms documented, n (% total)	APR (%)	Proportion of antibiotic prescriptions guideline adherent ^a^(%)
Primary care	182	77 (42.3%)	70/182 (38.5%)	23/70 (32.8%)
Chronic	99	66 (66.7%)	10/99 (10.1%)	5/10 (50.0%)
Acute	83	11 (13.3%)	60/83 (72.3%)	18/60 (30.0%)
Urgent care	100	3 (3.0%)	69/100 (69.0%)	40/69 (58.0%)
Chronic	26	1 (3.8%)	15/26 (57.7%)	11/15 (73.3%)
Acute	74	2 (2.7%)	54/74 (73.0%)	29/54 (53.7%)

APR, antibiotic prescribing rate.

a Total number of sinusitis encounters in which antibiotics were prescribed that met Stanford Guidelines Criteria for immediate or delayed prescription. Please see reference 8 for complete Stanford Sinusitis Guidelines. Acute ICD-10s: J01.00, J01.01, J01.10, J01.11, J01.20, J01.21, J01.30, J01.31, J01.40, J01.41, J01.80, J01.81, J01.90, J01.91. Chronic ICD-10s: J32.0, J32.1, J32.2, J32.4, J32.8, J32.9.

More antibiotic prescriptions were guideline adherent in urgent care compared to primary care encounters (58.0% vs 32.8%) (Supplemental Figure 1, Table 2). Antibiotics for sinusitis encounters were more frequently guideline adherent in the urgent care setting for both acute and chronic sinusitis encounters (Table 2).

## Discussion

We found that different ICD-10 definitions impacted the sinusitis APR, with higher rates observed for acute sinusitis ICD-10s compared to chronic ICD-10s. This difference is important because of the possible diluting impact of chronic sinusitis ICD-10s on the overall sinusitis APR.

The overall sinusitis APR was lower in primary care clinics; however, this is likely because chronic sinusitis ICD-10s were used more frequently in primary care sites. Notably, on chart review, chronic sinusitis in primary care encounters were less likely to have an antibiotic prescribed and commonly lacked documentation of active symptoms. This suggests the chronic sinusitis ICD-10s are often non-active and may be “carryover” ICD-10s from a previous encounter. Unlike urgent care encounters which are typically focused on one or two acute problems, primary care encounters are usually more comprehensive in which multiple issues may be addressed leading to more complex ICD-10 coding of both active and non-active problems. The lack of documentation of active symptoms for chronic sinusitis encounters in primary care poses challenges in accurately assessing antibiotic appropriateness. Understanding the defaults used within each institution’s EHR and leveraging it by creating templated documentation forms, order sets or other prompts may help abrogate this finding.

We found that despite a higher overall sinusitis APR in urgent care encounters, antibiotics prescribed were commonly guideline adherent. Truitt et al.^10^ found an APR of 80% for acute sinusitis in primary care clinics and on review found that 50% were appropriate. Our findings for acute sinusitis were similar; however, in their study, chronic sinusitis diagnoses were specifically excluded. Stenehjem and colleagues^5^ described the use of a similar tiered methodology to measure baseline and post-intervention antibiotic use in urgent care settings, including of a composite grouping of otitis media, sinusitis and pharyngitis, but did not examine sinusitis alone nor assess differential impact of acute versus chronic ICD-10s. This work and ours suggests that there is still significant overprescribing of antibiotics for sinusitis in the outpatient setting, reinforcing that this is an important target for antimicrobial stewardship.

Our project had several limitations. First, this was a single-center experience, which limits generalizability. Second, we did not quantify all possible opportunities in antibiotic prescriptions including choice, dose, and duration. Third, documentation may not always reflect the full array of symptoms a patient discussed during an encounter and billing data may be inaccurate. Fourth, despite the use of a tiered system for categorization, sinusitis may not always be the intended diagnosis for an antibiotic prescription.

Our findings are important for stewardship programs to consider when developing internal metrics for sinusitis to track the impact of interventions. The dilutional effect of combining acute and chronic ICD-10 in APR analysis for sinusitis highlights the need for improved ICD-10 utilization and methods to control for “carryover” ICD-10s. Until strategies are implemented to control for “carryover” ICD-10s of chronic problems in primary care, it would be prudent to analyze acute and chronic sinusitis ICD-10 categories separately to ensure that APR accurately reflects antibiotic prescribing practices.

## Supporting information

Smith et al. supplementary material 1 Smith et al. supplementary material

Smith et al. supplementary material 2 Smith et al. supplementary material

## Supplementary material

The supplementary material for this article can be found at https://doi.org/10.1017/ice.2024.200
