# Supplementary material for: No “One-Size-Fits All”: chronic “Carryover” diagnoses dilute antibiotic prescribing rates for sinusitis among adults in primary and urgent care settings
Source: Infect Control Hosp Epidemiol. 2024 Dec 27;46(2):203–5. doi: 10.1017/ice.2024.200 (PMC11790326; doi:10.1017/ice.2024.200)
Supplement: Smith et al. supplementary material 1 — Smith et al. supplementary material [file S0899823X24002009sup001.docx]

**Supplemental Figure 1. Antibiotic Prescribing for Sinusitis (Manual Chart Review 1/2021-12/2021)**

Supplemental Figure 1: Stacked bar chart depicting the percentage of urgent and primary care antibiotic prescriptions for acute and chronic sinusitis. Blue bars representing guideline-concordant prescriptions; orange bars indicated guideline non-concordant prescriptions, and grey bars denote encounters without antibiotic prescriptions.
