# Supplementary material for: No “One-Size-Fits All”: chronic “Carryover” diagnoses dilute antibiotic prescribing rates for sinusitis among adults in primary and urgent care settings
Source: Infect Control Hosp Epidemiol. 2024 Dec 27;46(2):203–5. doi: 10.1017/ice.2024.200 (PMC11790326; doi:10.1017/ice.2024.200)
Supplement: Smith et al. supplementary material 2 — Smith et al. supplementary material [file S0899823X24002009sup002.docx]

**Supplemental Table 1: Complete List of Urgent and Primary Care Clinics Sinusitis Encounter ICD-10s (1/2021-3/2022)**

| **Sinusitis (all ICD10s included)** | | **Total Encounters^¥^, n (% total)** |
| --- | --- | --- |
| **J01.00** | **Acute maxillary sinusitis, unspecified** | 222 (22.5%) |
| **J01.01** | **Acute recurrent maxillary sinusitis** | 47 (4.8%) |
| **J01.10** | **Acute frontal sinusitis, unspecified** | 100 (10.1%) |
| **J01.11** | **Acute recurrent frontal sinusitis** | 23 (2.3%) |
| **J01.20** | **Acute ethmoidal sinusitis unspecified** | 14 (1.4%) |
| **J01.21** | **Acute recurrent ethmoidal sinusitis** | 2 (0.2%) |
| **J01.30** | **Acute sphenoidal sinusitis unspecified** | 1 (0.1%) |
| **J01.31** | **Acute recurrent sphenoidal sinusitis** | 0 (0.0%) |
| **J01.40** | **Acute pansinusitis, unspecified** | 16 (1.6%) |
| **J01.41** | **Acute recurrent pansinusitis** | 13 (1.3%) |
| **J01.80** | **Other acute sinusitis** | 11 (1.1%) |
| **J01.81** | **Other acute recurrent sinusitis** | 3 (0.3%) |
| **J01.90** | **Acute sinusitis, unspecified** | 183 (18.5%) |
| **J01.91** | **Acute recurrent sinusitis unspecified** | 38 (3.9%) |
| **J32.0** | **Chronic maxillary sinusitis** | 45 (4.6%) |
| **J32.1** | **Chronic frontal sinusitis** | 15 (1.5%) |
| **J32.2** | **Chronic ethmoidal sinusitis** | 2 (0.2%) |
| **J32.4** | **Chronic pansinusitis** | 24 (2.4%) |
| **J32.8** | **Other chronic sinusitis** | 16 (1.6%) |
| **J32.9** | **Chronic sinusitis, unspecified** | 212 (21.5%) |

^¥^Total number and percent of tier 2 urgent or primary care clinic sinusitis encounters in which specific ICD-10 code was used
